# Surface Relief Grating on Chitosan-N,N-dimethyl-4-(2-pyridylazo)aniline Thin Film

**DOI:** 10.3390/polym14040791

**Published:** 2022-02-18

**Authors:** Nadiyah Rashed Al Atmah, Willian R. Caliman, Agnieszka Pawlicka, Ribal Georges Sabat, Jean-Michel Nunzi

**Affiliations:** 1 Department of Chemistry, Queens University, Kingston, ON K7L 3N6, Canada; 14nraa@queensu.ca; 2 Instituto de Química de São Carlos, Universidade de São Paulo, Av. Trabalhador Sãocarlense 400, São Carlos 13566-590, SP, Brazil; willian_caliman@usp.br (W.R.C.); agnieszka@iqsc.usp.br (A.P.); 3 Department of Physics and Space Science, Royal Military College of Canada, Kingston, ON K7K 7B4, Canada; sabat@rmc.ca; 4 Department of Physics, Engineering Physics and Astronomy, Queens University, Kingston, ON K7L 3N6, Canada

**Keywords:** chitosan, azo-dye, thin film, surface relief grating, hydrogen bond, naturally sourced polymer

## Abstract

We deposited homogeneous, thin, yellow-colored films of chitosan (Chi)-N,N-dimethyl-4-(2-pyridylazo)aniline (PADA) dye from an acid Chi–PADA solution by spin-coating on glass substrates. We characterized Chi, PADA, and Chi–PADA films by ATR–FTIR spectroscopy, which revealed a slight shift of 3170 and 3268 cm^−1^ bands, indicating H-bonding between the chitosan hydroxyl (OH) group and the amine (N) of the PADA pyridine ring. Based on these analyses, it was possible to determine the efficiency of the hydrogen bonds to form a Surface Relief Grating (SRG) on azo-polymer thin film. Moreover, we performed UV–VIS spectroscopy analysis of this film, which showed a broad band extending from 400 to 700 nm, with the maximum occurring at 428 nm. Therefore, we selected, within the absorption band, the 532 nm green laser wavelength to irradiate the azo-polymer films at room temperature. For the first time, natural polymer derivative and dye sample Chi–PADA thin films showed unique photoresponsive behavior under irradiation with two interfering laser beams. This permitted us to generate surface inscription patterning known as an SRG, which we confirmed by atomic force microscopy (AFM) and for which we determined a grating depth up to 50 nm. The present study opens the new possibility of using natural polymer-dye thin films.

## 1. Introduction

Over recent decades, azobenzene dye compounds have been investigated because of their photoreactive behavior when exposed to certain intensities of light sources [1]. This property opened new possibilities to explore them for practical applications such as non-linear optical devices, photo switching [2], nanoimaging, and optical data storage [3]. Three main azobenzene types differ from each other by their absorption spectra, which range from ultraviolet to visible red, so they can display three different colors: yellow, orange, and red for azobenzene, amino azobenzene, and pseudo-stilbenes, respectively [4]. When these chromophores are irradiated with a laser beam at frequencies within their absorption band, they undergo photoisomerization, photo reorientation, and mass movement, which result in a morphological deformation [5]. The same happens with polymeric azobenzene, which undergoes photoinduced *cis-trans-cis* isomerization [6]. Recently and for the first time, we showed that this reversible *trans-cis* isomerization occurs in Disperse red 1 (DR1) dye–hydroxypropyl cellulose (HPC) thin coatings, where the azo-dye compound was dispersed in a natural polymer derivative [7]. In this contribution, we showed that azo-dye could be hydrogen- or halogen-bonded to a natural polymer host and form a stable photo-activated material, similarly to other synthetic polymers [8]. However, in some other azo-dye polymer thin films, it is possible to produce a sinusoidally modulated surface, which is known as a Surface Relief Grating (SRG). This happens when a thin film of azobenzene material is irradiated with a light interference pattern [3,9,10], forming a high diffraction efficiency. Light polarization and intensity modulations are recorded on the thin film as microscopic morphological changes. These SRGs are stable at room temperature and can be erased by heating the sample to its glass transition temperature (Tg) [11,12].

Many studies have focused on understanding the process and the factors that influence the formation of SRGs, which depends on light polarization, the chemical structure of the material, and the photoinduced phenomena. However, a comprehensive understanding of the process and its mechanisms is still needed. Similar to our previous study on DR1-HPC, in the present study, we focus on azo-dye-doped polymer. Thus, azobenzene is not covalently attached to the polymer chain, but only forms noncovalent bonds, which are usually H-bonds. Therefore, the photoisomerization movement of the chromophores is not negatively affected by the polymer chain, and here the polymer chain migration is more likely the reason for the SRG [13].

Chitosan is a partially deacetylated chitin, i.e., poly(β-(1-4)-N-acetyl-D-glucosamine), which is a pseudo natural cationic polymer, soluble in aqueous acidic media, if deacetylation is at least 50% [14]. A protonation of new amine (–NH_2_) groups on the C-2 position of D-glucosamine rings is responsible for the solubilization of chitosan. Chitosan is interesting because of its low cost, biodegradability, and non-toxicity, so it presents promising potential in the food industry, and the pharmaceutic and bio-medical sectors [15]; for example, in wound healing management because it possesses antibacterial, anti-inflammatory and antioxidant properties [16], or in devices that include electrochromic windows [17,18]. Besides these, other chitosan advantages are that the aromatic rings help maintain its thermal stability and its functional hydroxyl and amine groups are susceptible to chemical modifications [4]. This leads to obtaining a great variety of derivatives that can be applied, for example, as promising chelating coagulants for several metal ions that pollute our environment [19].

A surface relief grating (SRG) is defined as a two-dimensional and periodic structure on the material surface that has dimensions of the wavelength used to engrave or monitor it. This kind of structure can be obtained by nanoimprinting, diamond turning, electron beam lithography, and reactive ion beam etching in dielectric materials [20], or can be photoinduced on samples of sol–gel, liquid–crystalline, or amorphous polymeric matrices doped with azo-dye, as in the case of poly(methyl methacrylate) containing Disperse red 1. The SRG’s unique properties were already applied with success in optical systems that include spectrometers, diffractive lenses, 3D pulse amplification, and scanning systems, and it has a great potential to be applied in photonics as optical memories or 2D planar photonic microstructures used as filters or couplers [21].

This report shows, for the first time, the results of SRG on azobenzene natural-polymer-derivative thin films by forming hydrogen bonds between the azobenzene and the polymer. An interference light pattern was used as the light source to irradiate the samples of azobenzene–polymer film and produce the gratings.

## 2. Materials and Methods

### 2.1. Chitosan–PADA Solution Preparation

An amount of 0.0134 g (0.01 mol/L) of chitosan (Chi; Sigma-Aldrich, Oakville, ON, Canada; medium molecular mass; 75–85% deacetylated) and 0.0452 g (0.02 mol/L) of N,N-dimethyl-4-(2-pyridyl-azo)aniline (PADA; 98%, Alfa Aesar, Haverhill, MA, USA) were separately dissolved in 3 mL of acetic acid solution with pH ≥ 2.3 (≥99.5%, Sigma-Aldrich, Oakville, ON, Canada and 99.8%, Merck, Rio de Janeiro, Brazil) under magnetic stirring for 48 h for complete dissolution. The acetic acid solution was prepared with Milli-Q^®^ water. After that, the PADA solution was poured into the chitosan solution and mixed and stirred for a further 24 h, resulting in a homogeneous solution of 1:2 mole Chi–PADA, which was used for film deposition on optical microscope glass substrates.

### 2.2. Chitosan–PADA Film Deposition

**Figure 1:**
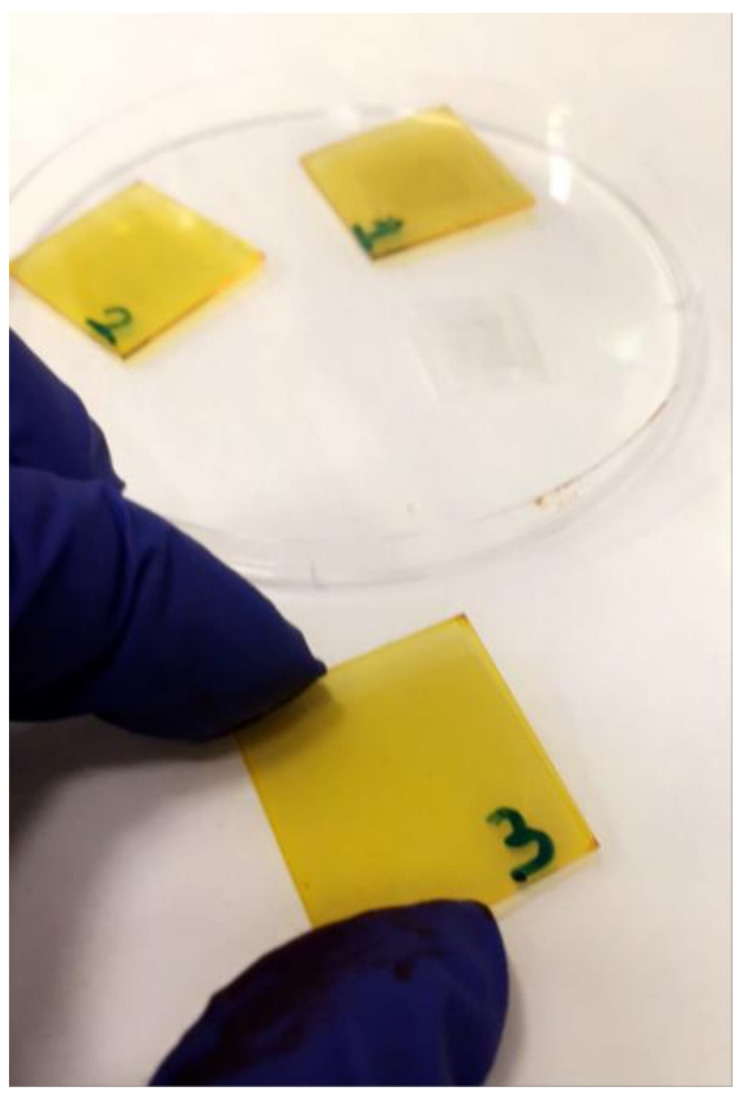
Picture of glass substrates coated with thin layer films of Chi–PADA.

The flat glass substrates (20 mm × 20 mm) were washed with water, soap, distilled water, isopropanol, and finally in acetone in an ultra-sonication bath for 10–15 min in each solvent. Then, they were dried by air dryer. A few drops of the chitosan–PADA solution were dripped onto the glass substrates and placed by a spin-coating technique with a spin coater (Laurell model Ws-400B-6NPP/LITE, North Wales, PA, USA), which was turned on at a spinning rate of 1000 rpm for 40 s [13]. After that, the films were removed from the spin-coater and thermally treated at 125 °C (Tg) for 10 min, resulting in homogenous, yellow-colored thin films (Figure 1).

### 2.3. Characterization of the Chi–PADA Films

The UV–VIS spectra of the Chi–PADA films were collected with a Hewlett Packard 8452A Diode Array (Canada) and/or JASCO 670 (Brazil) Spectrophotometers that operated in absorbance mode in a wavelength range between 300 and 800 nm.

**Figure 2:**
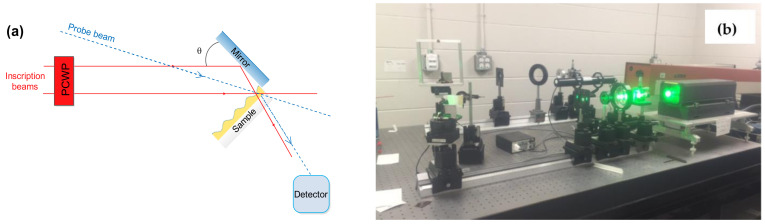
Laser setup for inscription of surface relief grating on Chi–PADA thin film: scheme (**a**) and picture (**b**). The SRG system is composed of two inscription and one probe beams, sample, mirror, detector, and a polarization-controlling wave plate (PCWD). Scheme was inspired by [4].

SRGs were fabricated using an Yttrium Aluminum Garnet (YAG) green laser (Verdi V5, 5W, Coherent, Mississauga, ON, Canada) set-up available in the laboratory, with a wavelength of 532 nm and operating at an irradiance of 1.5 W/cm^2^. The films were irradiated for 600 s at an incidence angle that resulted in 500 nm of grating spacing. The light interference pattern was obtained by adjusting the incident beam to split equally into two half beams. The first half of the beam was directly exposed on the surface of the film, while the other half was exposed on the film’s surface after reflection on a mirror to reflect it at the correct angle (*θ*). Both beams met at the surface of the film. The pitch (Λ) of the resulting grating was calculated from Equation (1).

(1) $$\wedge =\frac{\lambda }{2\sin\left(\theta \right)},$$

where *λ* is the wavelength of the incident light. The laser setup is shown in Figure 2.

**Figure 3:**
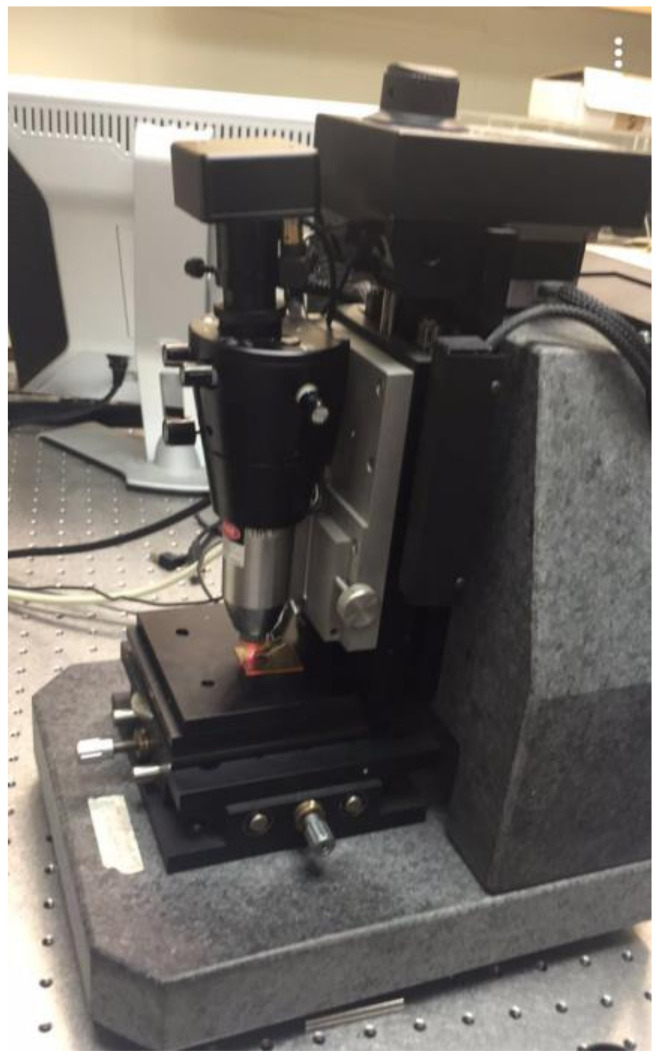
AFM measurements setup.

Atomic Force Microscopy (AFM) images of the Chi–PADA films were produced using Ambios Technology (Model No. EIU, 0927401, Santa Cruz, CA, USA) (Figure 3). For this, we employed the non-contact taping mode by using silicon AFM probes, a force constant of 48 N/m, and a resonance frequency of 190 kHz. All reported experiments were repeated 3 times for the optimum sample concentration, as well as the laser power level and irradiation time.

The FTIR (Bruker Alpha, Billerica, MA, USA) spectra of the Chi–PADA films were collected in attenuated total reflection (ATR) mode on the KBr pellet samples.

## 3. Results and Discussion

**Figure 4:**
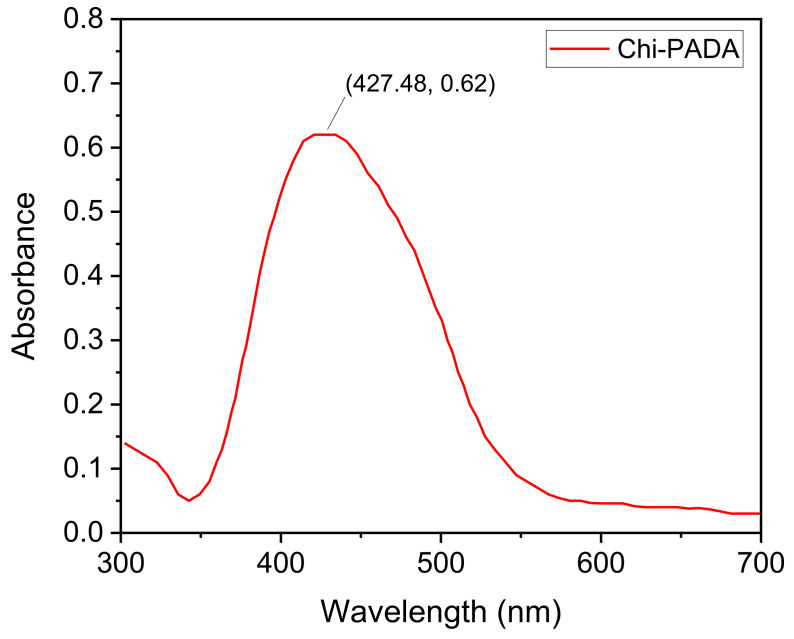
Representative UV–VIS spectrum of Chi–PADA thin film.

The Chi–PADA thin films (Figure 1) were characterized by UV–VIS and the obtained result is shown in Figure 4. This absorption spectrum shows a peak of *λ*_max_ at 428 nm, which corresponds to n-π* and π-π* transition. The UV–VIS spectrum for Chi–PADA was not recorded after the irradiation of the film by the green laser light due to the aggregation that occurred inside the film.

**Figure 5:**
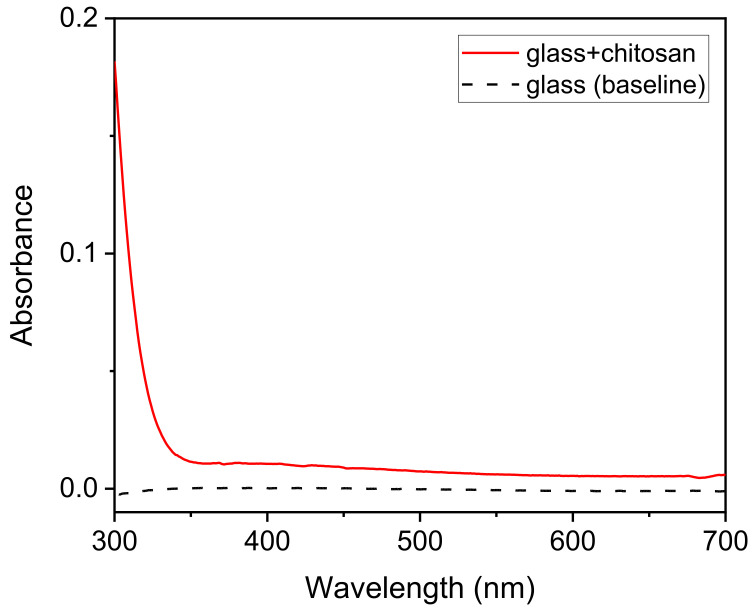
Representative UV–VIS spectrum of glass–Chi thin film and glass baseline.

To be sure that the absorbance band at 428 nm belonged to PADA, the glass substrate and chitosan film on the glass substrate were also analyzed. Chitosan has two chromophoric groups in its structure: N-acetylglucosamine and glucosamine, which absorb at about 225 nm (GlcN) [22], and this was also observed for our Chi solutions. However, as already expected, the glass–Chi sample did not show any absorption peak in the range of 300 to 700 nm (Figure 5). Therefore, the small shoulder observed from 300 to 350 nm in the Chi–PADA sample (Figure 4) is due to the glass–Chi contribution and the band centered at 428 nm to PADA.

**Figure 6:**
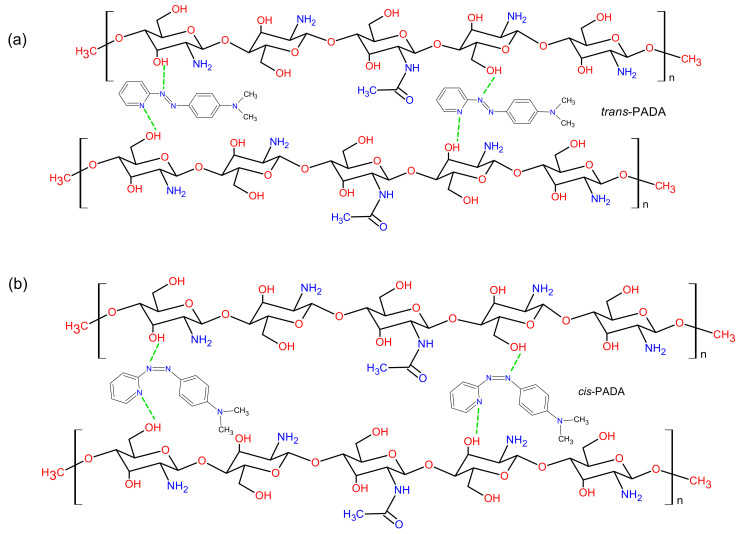
Representation of possible H-bonding between chitosan and PADA in its *trans* (**a**) and *cis* (**b**) forms.

The chemical structures of both Chi and PADA are shown in Figure 6. It is well known that chitosan has polar (OH and NH_2_) functional groups that can act as electron donors or interact with inorganic salts [23]. These groups can also form H-bonds with the azobenzene (PADA). On the other hand, the chemical structure of PADA indicates that the N in the pyridine ring can react as an acceptor, so together with OH donor groups of Chi, they can form a donor–acceptor system. The possibility of H-bonding was already observed in layer-by-layer self-assembled thin films of chitosan and cholesterol [24]. However, in their contribution, the authors discussed the possibility of the influence of pH on this kind of bonding because, in acid conditions, amine (NH_2_) Chi groups are protonated, which provides a solubility property to this natural macromolecule derivative. Nevertheless, a more recent contribution by Krajewska et al. [25] shows that the pH does not affect cholesterol−chitosan hydrogen bond formation in Langmuir monolayers, which is also probably valid in the present study.

**Figure 7:**
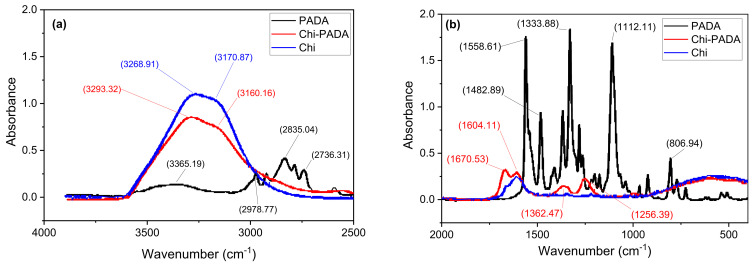
FTIR spectra of Chi (**–**), PADA (**–**), and Chi–PADA (**–**) in: (**a**) 4000–2500 cm^−1^; (**b**) 2000–400 cm^−1^ spectral ranges.

We used FTIR spectroscopy to examine the existence of hydrogen bonds in thin Chi–PADA films. The FTIR was individually measured for Chi and PADA, and then it was measured for Chi–PADA film in the KBr pellet samples (Figure 7). The broad band in pure Chi at 3171 cm^−1^ is associated with OH stretching, which overlaps with NH stretching at 3269 cm^−1^. As this band is large, we assigned it to intra- and intermolecular hydrogen bonding including the OH groups [26]. Of the two bands at 1604 and 1670 cm^−1^, the former can be attributed to chitosan NH_2_ and the latter is reminiscent of chitin amide I (C=O stretching vibration) [26].

The FTIR of PADA has many absorbance peaks. The peak at 807 cm^−1^ can be attributed to Ar-H; at 1112 cm^−1^ to the vibration of the C-N bond in the pyridine ring; at 1334 and 1483 cm^−1^ to CH_3_ symmetric and asymmetric bending overlapped with –N=N– vibration; at 1559 cm^−1^, it is due to the benzene ring; and at 2736 to 2979 cm^−1^ to CH aliphatic and aromatic stretching. The large peak at 3365 cm^−1^ is probably due to a small amount of water being absorbed.

The FTIR spectrum of Chi–PADA is very similar to the FTIR spectrum of Chi, but the absorbance of the Chi–PADA complex shows a change in the intensity of the bands at 3170 and 3268 cm^−1^ and their slight shift (stretching) to 3293 and 3160 cm^−1^ [27]. This change can be caused by the H-bonding formation between the hydroxyl (OH) group in Chi and the nitrogen (N) atoms in the pyridine ring of PADA (Figure 5). Additionally, the bands of Chi–PADA at 1670 and 1604 cm^−1^ increased and two new bands appeared at 1362 and 1256 cm^−1^, probably due to the interaction between chitosan and PADA.

**Figure 8:**
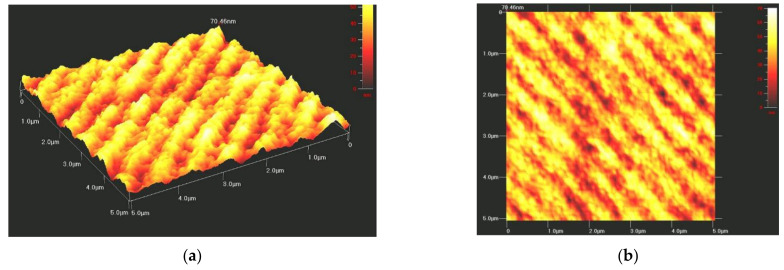
(**a**) Three-dimensional and (**b**) two-dimensional AFM images of Chi–PADA SRG.

Besides a few optical studies on rare-earth atoms doped Chi [28,29], this matrix is still a new material, so this contribution aimed to advance the field by applying this polymer to optical studies. Similarly to other azo-polymers [21], the Chi–PADA samples were submitted to interferential beam irradiation to observe the formation of surface relief gratings (SRGs). This was possible by scanning them after laser beam exposure with an Atomic Force Microscope (AFM) in the tapping mode. Figure 8 shows AFM images for a thin film of Chi–PADA-recorded SRG. The sinusoidal grating is well visible, with regular spacing of about 500 nm and depths of around 49 nm. The experiment was repeated many times with different irradiation times, selected spacings, and power levels. The reported result is the largest grating that we obtained in the films. The obtained gratings were stable for six months. Finally, we observed that efficient *trans-cis* isomerization–polymerization plays a role in grating formation and the thermal lifetime of the grating.

## 4. Conclusions

For the first time, we produced a smooth film of the natural polymer, chitosan, and azobenzene dye, N, N-dimethyl-4(2-pyridylazo)aniline. This film showed a broad absorption spectrum with a peak maximum of *λ*_max_ at 428 nm, which corresponds to n-π* and π-π* transition. The FTIR spectra of the Chi–PADA complex revealed a change in the intensity of the bands at 3170 and 3268 cm^−1^ and their slight shift to 3293 and 3160 cm^−1^, interpreted as the H-bonding formation between Chi hydroxyl (OH) groups and the nitrogen (N) atoms of PADA pyridine rings. Chi–PADA bands at 1670 and 1604 cm^−1^ also increased and two new bands appeared at 1362 and 1256 cm^−1^, probably due to the interaction between chitosan and PADA. Finally, this film was irradiated with two interfering green laser beams of 532 nm, which revealed its unique photoresponsive behavior. This property permitted us to generate surface inscription patterning known as surface relief grating. We confirmed the SRG formation by atomic force microscopy, which was also used to see the sinusoidal grating with about 500 nm regular spacing and a grating depth of up to 50 nm. The gratings were stable for six months in normal conditions, which corroborated the hydrogen bond formation between the =N- in the pyridine ring of azo-dye and the OH of chitosan. In summary, these H-bonds were a sufficient link to inscribe an SRG.

## Data Availability

Not applicable.
